# Q&A: What are pathogens, and what have they done to and for us?

**DOI:** 10.1186/s12915-017-0433-z

**Published:** 2017-10-19

**Authors:** Francois Balloux, Lucy van Dorp

**Affiliations:** UCL Genetics Institute (UGI), Darwin Building, Gower Street, London, WC1E 6BT UK

## Abstract

Microbes are found on us, within us and around us. They inhabit virtually every environment on the planet and the bacteria carried by an average human, mostly in their gut, outnumber human cells. The vast majority of microbes are harmless to us, and many play essential roles in plant, animal and human health. Others, however, are either obligate or facultative pathogens exerting a spectrum of deleterious effects on their hosts. Infectious diseases have historically represented the most common cause of death in humans until recently, exceeding by far the toll taken by wars or famines. From the dawn of humanity and throughout history, infectious diseases have shaped human evolution, demography, migrations and history.

## What is a pathogen?

A pathogen is defined as an organism causing disease to its host, with the severity of the disease symptoms referred to as virulence. Pathogens are taxonomically widely diverse and comprise viruses and bacteria as well as unicellular and multicellular eukaryotes. Every living organism is affected by pathogens, including bacteria, which are targeted by specialized viruses called phages.

The number of viruses and bacteria on earth is staggering and they occupy essentially every environment. A liter of surface seawater typically contains in excess of ten billion bacteria and 100 billion viruses. The number of viruses on Earth is estimated to be around 10^31^, which corresponds to roughly ten billion times the number of stars in the universe [1]. An average human is made up of about 30 trillion cells but carries a similar number of bacteria, mostly in the gut [2].

The vast majority of viruses and bacteria we are exposed to have no negative effect and some can even be beneficial, though a tiny fraction of these can severely affect our health. Specifically, about one in a billion microbial species is a human pathogen. Indeed, approximately 1400 human pathogens have been described, whereas it has been estimated that there are one trillion microbial species on Earth, the vast majority of which remain uncharacterized [1].

## What is the relationship between pathogens and hosts?

Pathogens can be divided into two main categories, namely facultative and obligate pathogens, reflecting how intimately their life cycle is tied to their host.

Facultative pathogens are organisms for which the host is only one of the niches they can exploit to reproduce. Facultative pathogens are primarily environmental bacteria and fungi that can occasionally cause infection. They include many of the most problematic hospital-acquired bacteria involved in the antimicrobial resistance pandemic. A distinction is sometimes made between facultative and accidental pathogens, with the latter representing those which only occasionally infect weakened or immunocompromised hosts. Typical examples of ‘accidental’ pathogens include *Neisseria meningitidis* or *Escherichia coli*.

Obligate pathogens require a host to fulfil their life cycle. All viruses are obligate pathogens as they are dependent on the cellular machinery of their host for their reproduction. Obligate pathogens are found among bacteria, including the agents of tuberculosis and syphilis, as well as protozoans (such as those causing malaria) and macroparasites.

Some obligate pathogens require multiple different hosts to fulfil their life cycle. The definite host, which supports the adult form of the pathogen, is often a vertebrate and the intermediate host (referred to as a vector) is generally an arthropod or a mollusc. This alternation of vertebrate and invertebrate hosts is found in viruses (for example the Zika virus), bacteria (for example Lyme disease) and protozoa (malaria). Trematodes (parasitic flatworms) go even further and some exhibit among the most baroque life cycles. Digenetic trematodes have a basic three-host life cycle, and for some species a four-host life cycle. For instance, *Halipegus occidualis* sequentially has to infect a freshwater snail, an ostracod, a dragonfly nymph and ends its cycle after the dragonfly is eaten by the green frog *Rana clamitans*, where it resides under its tongue [3].

## What is the host range of pathogens?

Some pathogens are limited to infecting a single host species, whereas others can infect a multitude of host species. Host ranges can feel highly idiosyncratic if not outright puzzling. For example, leprosy in humans is caused by two related intracellular bacteria *Mycobacterium leprae* and *Mycobacterium lepromatosis*, which are essentially restricted in the wild to humans, as well as armadillos in the Americas and red squirrels in Scotland [4].

Conversely, *Yersinia pestis*, another intracellular obligate bacterium and the agent of plague, has a natural life cycle involving alternating infections of rodents and fleas, but can infect essentially any mammalian host. An interesting twist in the case of plague is that *Y. pestis* is not well adapted to the human host. With the exception of uncommon occurrences of human-to-human transmissions, referred to as pneumonic plague, plague epidemics (bubonic plague) are caused by plague-infected fleas biting humans. Somewhat ironically for a pathogen that is possibly the biggest killer in human history, bubonic plague is a complete evolutionary disaster. The human host is at a very high risk of dying, the flea cannot reproduce on a meal of human blood and the bacterium is stuck in an evolutionary dead-end as it cannot transmit to another host.

There is no obvious predictor for the host range of different pathogens. Intuitively, it may be tempting to predict that pathogens with a more intimate relationship with their host are more closely adapted to their host, and thus have a more restricted host range. However, there is no obvious pattern suggesting that viruses (that rely on the host cells’ machinery for reproduction) have a narrower host range than bacteria. Also, intracellular bacteria do not seem to have a markedly narrower host range than extracellular ones, despite being more intimately tied to their host.

We know relatively little about the underlying genetic changes required for a pathogen to infect a new host, though, interestingly, only a few mutations can be required for a host jump. For example, avian influenza is only around five mutations away from being able to transmit in mammals [5], and a single amino acid change was sufficient for the human-adapted bacterium *Staphylococcus aureus* to become a pathogen of rabbits [6].

## What is special about pathogen genomes?

Obligate pathogens tend to be highly adapted to their hosts, with sophisticated mechanisms to synchronise their life cycles with that of the host, and the ability to manipulate the host’s immune system, metabolism and sometimes even behaviour. Genes encoding proteins specific to pathogenicity are referred to as virulence factors, which include a variety of molecules required for colonization of the host, immunoevasion and immunosuppression, scavenging nutrients within the host, and entry into and exit out of cells for intracellular pathogens.

In bacteria, virulence factors are often found in groups of genes on pathogenicity islands, which can be transferred horizontally by plasmids or other transposable elements. For example, one of the defining features of the plague bacterium *Y. pestis* from its less virulent closest relative *Yersinia pseudotuberculosis*, is the inclusion, early in its evolution, of two plasmids carrying genes involved in pathogenicity [7].

While acquisition of novel genes and repurposing of existing ones is essential in the evolution towards pathogenicity, a general feature during the evolution towards pathogenicity is genome reduction through the inactivation and loss of genes. This can be primarily explained by the fact that a host represents a fairly stable and resource-rich environment where some metabolic pathways required in the environment are not necessary. Genome reduction is a general trend accompanying the evolution towards pathogenicity and is observed in *Mycobacterium tuberculosis*, pathogenic *E. coli* strains and in the ongoing adaptation of *Klebsiella pneumoniae* lineages to cystic fibrosis patients. The most extreme example is leprosy (*M. leprae* and *M. lepromatosis*), which has shed nearly half the genes found in their environmental relatives [8]. Another interesting tendency of many bacterial pathogens is the secondary loss of the ability to undergo genetic recombination [9].

## How do pathogens cause disease?

Pathogens cause illness to their hosts through a variety of ways. The most obvious means is through direct damage of tissues or cells during replication, generally through the production of toxins, which allows the pathogen to reach new tissues or exit the cells inside which it replicated. Bacterial toxins are among the deadliest poisons known and include famous examples such as tetanus, anthrax or botulinum toxin, known as Botox in its commercial application.

However, the damage to the host is often self-inflicted through a strong or sometimes excessive immune response that indiscriminately kills infected and uninfected cells and damages host tissues. Typical examples of maladaptive over-reaction of the immune system include cirrhosis and liver cancer in hepatitis B [10], or the 1918–1919 influenza epidemic, where the toll was highest amongst the young and healthy possibly because they mounted the strongest immune response and as such died from a ‘cytokine storm’ in the lungs leaving patients literally drowning in their own body fluids [11].

Some pathogens benefit from the hosts’ immune reaction to spread within an infected host or increase their transmission to uninfected hosts. Influenza transmits mainly through aerosols created through the sneezing and coughing it causes. *Vibrio cholerae* triggers a strong inflammatory response in the gut mucosa, leading to watery diarrhoea and ensuring its release in the environment and thus infection of further hosts.

## Are co-evolved pathogens less virulent?

Pathogens greatly vary in the severity of their symptoms from a mild inconvenience to assured death. It is sometimes assumed that the deadliest pathogens represent recent host jumps where the pathogens’ virulence is maladapted to the new host, and that co-evolution between host and pathogen will lead to more benign symptoms over time. However, this is only true in the case of strict vertical transmission (such as from mother to child), where survival and transmission of host and pathogen are intimately linked.

In the case of horizontal transmission, the situation is more complex and there is no straightforward way to predict the evolution of future virulence, as it will depend on a variety of factors, including the population structure of the host and the correlation between virulence and transmission [12]. A textbook example for reduction in virulence is the introduction of myxomatosis into the European rabbit population in Australia and France in 1950 and 1952, respectively. Upon introduction, the virus initially killed about 99% of infected rabbits but over a few years mortality went down to 90%, following the emergence of attenuated virus strains and genetic resistance in the rabbit population. While the virulence went down, which translated into higher transmission rates, the current 90% mortality rate remains exceedingly high and there is no evidence for further reduction in the short term [13].

*Bd*GPL, the global lineage of the amphibian fungal pathogen *Batrachochytrium dendrobatidis* (*Bd*), is the only known pathogen to have extirpated entire host species. Yet, over the three decades since its discovery, it shows no sign of evolving lower virulence. The primary reason there is little selective pressure on *Bd* for virulence attenuation is that it can infect a very vast host range so that the extinction of any particular host species has limited impact on its fitness. Even worse, some host species, such as the widely introduced African clawed frog, *Xenopus laevis*, and the American bullfrog, *Rana catesbeiana*, carry the disease asymptomatically, fuelling the global *Bd* pandemics and limiting any short-term prospect for significant decrease in virulence [14]. These are just examples of the evolution of virulence but both illustrate that there is no simple pattern of decrease in pathogenicity with time.

## How old are the major human pathogens?

Apart from a few putative ancestral pathogens, including *Helicobacter pylori* [15], that might have co-speciated with their human host, the infectious diseases afflicting us were acquired through host jumps from other wild or domesticated animal hosts or sometimes from the wider environment. The timing of these events and the original source remains unclear in many cases.

The traditional view has been that many human pathogens emerged during the Neolithic revolution. The main arguments for an origin of human pathogens linked to agriculture are based on the proximity between traditional farmers with their livestock and the emergence of higher human population densities in stable settlements enabled by agricultural subsistence. High population density is indeed required by some epidemic diseases which could not have maintained themselves on scattered groups of hunter-gatherers [12]. This argument, however, neglects the fact that pathogens can evolve fast. Also, while the proximity of humans and livestock is conducive to host jumps, humans transmitted more diseases to domestic animals than they acquired, with tuberculosis in particular having probably jumped from humans to cattle rather than the other way around [16]. Finally, this argument also neglects the high burden of pathogens in wild populations, including in the great apes.

**Fig. 1.**
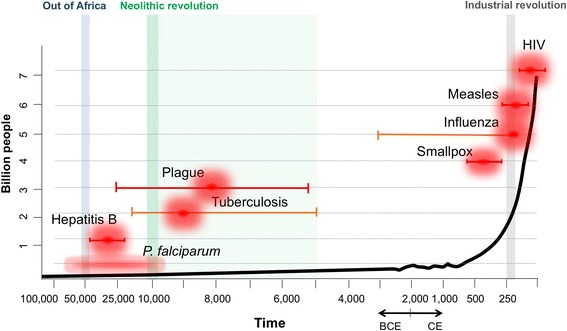
Age of emergence of significant infectious diseases impacting on human populations. The time of emergence for the various major diseases is based on a synthesis of published research. When known with some confidence, point estimates are provided for each disease together with error bars depicting uncertainty in the inferred estimates. *Orange error bars* depict higher uncertainty compared to *red*. The *black trend line* plots an increase in human population size through time (*x axis*) in the order of billions of people (*y axis*). Key events in human history are highlighted and annotated at the top. Main references used were: smallpox [27]; influenza [28]; HIV [29]; tuberculosis [30–33]; *P. falciparum* malaria [34, 35]; hepatitis B [36]; measles [37]; plague [38, 39]

Ancient direct evidence is scant for pathogens, and historical records rarely allow unambiguous attribution of described symptoms to a disease. That being said, recent progress in sequencing technology and in particular the ability to generate sequences, if not complete genomes, from ancient samples has greatly improved our understanding of the age of the major human pathogens, often leading to unexpected results. Figure 1 summarises the current knowledge on the age of the seven current ‘major killers’, as well as plague, which was included due to its major impact in the past. While some of these estimates may need to be updated in the future after the emergence of new evidence, it is unlikely the general pattern will change much. Some human diseases are old (for example *Plasmodium falciparum* malaria) and others recent, such as HIV or, more surprisingly, measles. There is also no obvious pattern pointing to the Neolithic revolution as a strong driver for the emergence of human pathogens.

## Where are the resistance-conferring genes in the human genome?

Infectious diseases have killed well over half of all humans who have ever lived on earth. Pathogens, such as childhood diseases, that affect their host prior to reproduction, through death or reduced fertility, have exerted enormous selective pressures. Yet, scans of the genome for signatures of pathogen-driven selection have identified only a few variants with clear effects. Similarly, genome-wide association studies (GWAS) for infectious disease resistance/susceptibility have identified only few loci impacting on infectious disease susceptibility [17], despite their success in identifying thousands of variants involved in chronic diseases and phenotypic traits such as height. Even for diseases that have affected us for a long time, for example tuberculosis, we know of no obvious protective genetic variant.

Given the high selective pressure pathogens must have exerted, it is reasonable to ask where all the resistance genes are. Strongly protective variants may have reached fixation, rendering them undetectable unless the pathogen has a highly heterogeneous distribution range. An interesting case for regional selective pressure is the Duffy negative antigen mutation protective against *Plasmodium vivax* that is found at close to 100% in Sub-Saharan Africa but virtually absent anywhere else. Another situation where a resistance gene does not reach fixation arises when the protective variant is deleterious when homozygous, as in sickle cell anaemia. We might also speculate that the evolutionary potential and high genetic diversity of most pathogens limits our ability to detect protective variants in the human genome, particularly so if these were only effective against a subset of lineages within a pathogenic species.

In addition to the few variants protective against specific pathogens, we also know of genomic regions involved in immunity against a wide spectrum of pathogens, such as interleukin genes or the major histocompatibility complex (MHC) system. The very high genetic diversity of the MHC is believed to have been shaped by exposure to different pathogen species [18]. Also, following the recent development of techniques to sequence ancient DNA, it has been suggested that immunity genes such as those encoding toll-like receptors have been acquired following hybridization with archaic humans and are over-represented in the current gene pool of anatomically modern humans relative to genes not involved in immunity [19, 20].

## How did pathogens shape human history?

Infectious diseases had a massive impact on our history, leading to the rise and fall of civilizations, both through the toll they took on human life but also through economic and societal collapse following epidemics. More military campaigns have probably been lost or won due to infectious diseases than due to the tactical acumen of the armies’ commanders. Thucydides reports in his *History of the Peloponnesian War*, written in the 5^th^ century BC, how the plague of Athens devastated the city-state of Athens in ancient Greece during the second year of the Peloponnesian War (430 BC) when it was on the cusp of victory against Sparta, ending the golden age of Pericles and Athenian predominance in the ancient world. The eventual fall of the Roman Empire was also largely down to another epidemic, the Justinian plague in 541–542 CE, which precluded the Emperor Justinian recovering lost territories in the western part of the empire [21].

Infectious disease played an equally important role in past human migrations. The conquistadores’ sweeping conquests of large swaths of the Americas in the 16^th^ century was greatly aided by the diseases they brought with them, such as measles and smallpox, to which the indigenous populations had limited immunity [22]. Conversely, one of the possible reasons Europeans managed to colonize Africa was that they used quinine, an antimalarial drug derived from the bark of the cinchona tree [23].

History has been shaped not only by pathogens infecting humans, but also those affecting domestic animals and crops. For example, it has been suggested that the Islamic conquest of the 7^th^ and 8^th^ centuries did not extend to Sub-Saharan Africa because the horses and camels of the Islamic armies were dying from trypanosma spread by tsetse flies [24]. Conversely, pathogens were at other times the drivers of large migration. Around one million Irish people died and another million migrated to the US to escape the famine caused by *Phytophthora infestans* destroying potato harvests between 1845 and 1852 [25].

## But what about now?

At least in the developed world, the leading causes of human mortality are no longer infectious diseases but instead age-associated disorders such as cancer, heart disease and diabetes. Numerous countries have undergone an epidemiological transition, starting some 300 years ago in some developed countries and less than 80 years ago for developing countries. Diseases that once devastated human populations, such as smallpox, are now eradicated. Others, such as the plague or leprosy, are largely under control with the exception of a few hotspots.

The current situation is, however, one of new challenges. Globalization and increased mobility, particularly air travel, have facilitated the transmission of diseases not just locally but between continents. The recent outbreak of Zika in the Americas, for example, has been attributed in part to an increase in air travel from infected areas into Brazilian airports, extending both the incidence and geographic range of the virus [26]. The 2003 outbreak of severe acute respiratory syndrome (SARS) and recurrent Ebola crises in Central Africa highlight the ability of new and existing diseases rapidly to become significant international health threats. In addition, our ability to combat infectious diseases is also challenged by the widespread emergence of pathogen drug resistance. The global antimicrobial resistance (AMR) crisis is increasingly limiting our resources to combat disease through antimicrobial therapy.

Thus, in spite of the global health narrative supporting a decline in the number of deaths caused by infectious disease, the complexity of our interactions with disease-causing agents are as significant now as through history. Infectious diseases continue to be a major cause of mortality globally, responsible for between a quarter to a third of all deaths and nearly half of all deaths in people under the age of 45, with most of these in principle avoidable.
